# Supplementary material for: High-resolution kinetics of herbivore-induced plant volatile transfer reveal clocked response patterns in neighboring plants
Source: eLife. 2024 Feb 22;12:RP89855. doi: 10.7554/eLife.89855 (PMC10942584; doi:10.7554/eLife.89855)
Supplement: Supplementary file 1. [file elife-89855-supp1.docx]

**Supplemental file 1.** Gene identifiers and qRT-PCR primer sequences used in this study.

| **Target gene** | **Gene name** | **Identifier** | **fw** | **rv** |
| --- | --- | --- | --- | --- |
| TPS2 | Terpene synthase 2 | Zm00001d015053 | TACCGGGTCGAGATCACCAA | TCGTTCGTAACGGTGTGGAG |
| TPS10 | Terpene synthase 10 | Zm00001d024486 | TGACAGCCTTGATCACCGTA | AGTTCATCCCCACACGAATC |
| FPPS3 | Farnesyl pyrophosphate synthase 3 | Zm00001d043727 | CCTGGCTAGTTGTGCAAGCT | CAAAACAGTTTGGACTGCCT |
| CYP92C5 | Dimethylnonatriene/  trimethyltridecatetraene synthase | Zm00001d018839 | AGGGGTTCAAGCGGAAGATG | AGGTCAGTCGCCACAAACTC |
| OPR7 | Oxo-phytodienoate reductase 7 | Zm00001d032049 | GCACGCATGTGATTTGATTATTAGT | CAA TCG CGG CAT TAC CCA GAT GT |
| UBI1 | Ubiquitin | Zm00001d015327 | TAAGCTGCCGATGTGCCTGCG | CTGAAAGACAGAACATAATGAGCACAG |
